# Effects of an Exercise Intervention on Gait Function in Young Survivors of Osteosarcoma with Megaendoprosthesis of the Lower Extremity—Results from the Pilot Randomized Controlled Trial proGAIT

**DOI:** 10.3390/curroncol29100613

**Published:** 2022-10-14

**Authors:** Simon Basteck, Wiebke K. Guder, Uta Dirksen, Arno Krombholz, Arne Streitbürger, Dirk Reinhardt, Miriam Götte

**Affiliations:** 1 Department of Pediatric Hematology/Oncology, Clinic for Pediatrics III, West German Cancer Centre, University Hospital Essen, 45147 Essen, Germany; 2 German Cancer Consortium (DKTK), Partner Site Essen, 45147 Essen, Germany; 3 Department of Orthopedic Oncology, West German Cancer Center, University Hospital Essen, 45147 Essen, Germany; 4 Faculty of Sport Science, Ruhr University Bochum, 44801 Bochum, Germany

**Keywords:** bone tumor, AYA, endoprosthesis, exercise, gait analysis, lower limb function

## Abstract

Limb preservation with megaendoprosthesis in adolescents and young adults (AYA) with bone tumors is associated with functional limitations and gait abnormalities. The proGAIT trial evaluated the effectiveness of an exercise program on gait function and quality of life, functional scales (MSTS, TESS), functional mobility, and fatigue as secondary outcomes. Eleven AYA survivors of malignant osteosarcoma with a tumor endoprosthesis around the knee (mean age: 26.6 (±8.4) years) were randomized into an intervention group receiving an 8-week exercise program or into a control group. Gait function was assessed via 3D motion capture and analyzed using the Gait Profile Score (GPS) and the Gait Deviation Index (GDI). GDI and GPS scores of participants suggest deviations from a healthy reference group. The exercise intervention had small-to-medium positive effects on gait score GDI |d| = 0.50 (unaffected leg), |d| = 0.24 (affected leg), subjective functional scores TESS |d| = 0.74 and MSTS |d| = 0.49, and functional tests TUG and TUDS |d| = 0.61 and |d| = 0.52. None of these changes showed statistical significance. Promising intervention effects suggest that regular exercise could improve lower limb function and follow-up care for survivors; however, a powered RCT as a follow-up project needs to confirm the pilot findings.

## 1. Introduction

The incidence of bone tumors is particularly high in adolescents and young adults (AYA) between 15 and 39 years of age [1]. Due to the development of new treatment methods, improved imaging techniques determining the disease extent, and improved surgical techniques, the overall survival of patients is above 60% [2,3,4]. These improved survival rates are bringing the focus on addressing psychological and physical long-term consequences and late effects, and underline the need of supportive concepts to ameliorate these negative effects. These approaches include behavior change interventions in the areas of adequate physical activity, healthy diet, smoking cessation, and alcohol consumption [5,6]. Previous research has shown that the quality of life is reduced in survivors of bone tumors, and the prevalence of somatic disease and psychological problems is high in comparison to matched comparison groups [7,8]. Modular megaendoprostheses have become a gold standard in the reconstruction of osteoarticular defects following tumor resections, while amputation can be avoided in the majority of patients [9]. Limb salvage has both functional and psychological benefits compared to amputation of the affected limb. However, studies suggest that former bone tumor patients have specific gait and functional limitations [10]. These also affect the quality of life of this patient cohort and their participation in daily, social, and professional life, and sports [11,12,13]. The most frequently reported limitations are regular pain, difficulties in participation in sports and other everyday life activities [14]. Initial interventions indicate positive effects of rehabilitative and exercise programs on the postural control and walk ratio of patients with bone or soft tissue sarcomas [15]. In addition, high evidence levels indicate that exercise is beneficial for cancer patients and survivors in general to reduce fatigue, anxiety and depression, and increase physical performance, quality of life, and bone health [16,17]. The group of adolescent and young adult bone tumor survivors and the effects of exercise in this cohort have only been studied in very few trials. Winter et al. [18] evaluated an individualized exercise program and found that it was feasible, safe, and tended to be beneficial to increase physical activity levels. The lack of data on bone tumor patients contrasts with the high need and burden of this group. It is well known that young adults in the process of structuring their private and educational life have a very special need for supportive services [19]. Limited mobility in everyday life, and the presence of late effects, can severely limit sports participation and participation in social life [11]. However, standardized methods to monitor patients during follow-up, to analyze their gait pattern, and to evaluate the effectiveness of appropriate exercise programs have not yet been established. The main objective of this randomized controlled pilot trial was to investigate the effects of an individualized 8-week exercise program on gait function in adolescents and young adults (AYA) with megaendoprosthesis of the lower extremities in their follow-up care.

## 2. Materials and Methods

### 2.1. Design

The inclusion criteria were: (1) a confirmed lower extremity bone tumor (osteosarcoma, Ewing sarcoma, chondrosarcoma), (2) had follow-up care at the University Hospital Essen, (3) aged between 15 and 45 years, and (4) had implantation surgery at least 12 months prior to the baseline assessment. Exclusion criteria were medical conditions that preclude participation in the testing and/or the intervention. The inclusion age range was extended over typical AYA definition to allow for an expanded pool of potential participants. The study population characteristics are summarized in Table 1. Written informed consent was required to participate in the study and the local Ethics Committee of the Faculty of Sport Science at Ruhr University Bochum approved this study (reference number EKS V 04/2021). Recruitment was performed in the period 1 August–16 October 2021 by contacting former patients with megaendoprosthesis of the lower extremity.

The proGAIT study (NCT04963517) was a randomized controlled trial (RCT). Randomization was carried out via minimization using the software minimPy version 2.0 by Dr. M. Saghaei [20]. Factors in the randomized allocation process were participants’ resection length of the affected limb and participants’ age. Both factors had two levels. The shortest reconstruction lengths were 100 mm or 120 mm for the distal femur and 115 mm or 135 mm for the proximal tibia. All longer resections were reconstructed using extension sleeves in a modular implant design. Sleeves were available in 30 mm, 40 mm, 60 mm, 80 mm, and 100 mm. Combinations were also used. Based on this, a classification between the shortest possible (<140mm) and longer (≥140mm) resections/reconstructions for both localizations (distal femur, proximal tibia) was chosen. Different levels of age were classified with <30 years and ≥30 years. Participants were allocated to the study groups directly after their baseline visit.

### 2.2. Study Population and Surgery

All patients included in this study were reconstructed using a linked knee megaendoprosthesis (implantcast GmbH, Buxtehude, Germany, MUTARS system) following bone tumor resections of the distal femur or proximal tibia. Oncological tumor resections, disregarding the tumor site, aimed at the complete removal of the tumor that was surrounded by a healthy soft tissue margin, thus, in distal femur tumors that entailed the detachment and partial loss of the vastus medialis, vastus lateralis, vastus intermedius and adductor muscles. Both venters of the gastrocnemius muscle were severed close to their insertion in the distal femur. The patella and insertion of the patella tendon at the tuberosity of the tibia were retained. While the femoral insertion of both venters of the gastrocnemius muscle remained intact in proximal tibia resections, the patella ligament was detached from its insertion at the tibial tuberosity. The following muscles inserted at the proximal tibia were severed, leaving a margin of healthy muscle around the tumor: tibialis anterior, extensor digitorum and hallucis longus, soleus, tibialis posterior, flexor digitorum, and hallucis longus. An attachment tube covering the implant body of the proximal tibia megaendoprosthesis was used for the refixation of the patella ligament. Additionally, to improve the soft tissue coverage of the implant, patients were reconstructed using a local gastrocnemius flap.

However, since both the distal femur and proximal tibia resections were osteoarticular resections around the knee, they shared common characteristics as well: both cruciate and collateral ligaments needed to be severed, necessitating a reconstruction using a linked megaendoprosthesis. As a result, the long-term flexion of the knee joint was limited to 90° due to the metal-on-metal rotating-hinge coupling piece used in this patient collective.

Early rehabilitation included a period of six weeks with the partial weight bearing of 20 kg after the cementless implantation of megaendoprostheses of either site. However, while the knee joint flexion was increased by 30° each week immediately after distal femur resection, the refixation of the patella tendon and gastrocnemius flap on the attachment tube led to a four-week immobilization period of the knee joint using an extension brace. Thus, flexion was only increased by 30°, starting in week five after the surgery. Patients after proximal tibia replacement also commonly suffer from a weakness of dorsiflexion of the foot, which is supported by an ankle foot orthosis for several months after the operation until active dorsiflexion recovers. After the completion of adjuvant chemotherapy, all patients are eligible for a three-to-four-week inpatient rehabilitation program to recover socially, psychologically, and physically.

To control for different functional abilities and requirements during early rehabilitation after distal femur and proximal tibia resection, this study recruited patients only if their surgery was performed at least 12 months prior to the baseline evaluation. By that time, rehabilitation no longer needs to be site-specific, and varying site-specific early functional impairments had usually recovered. In addition, no spontaneous improvement without training was expected after 12 months [21].

### 2.3. Intervention

The intervention group (IG) received a personalized 8-week training consisting of exercises focusing on strength, coordination, balance, and mobility of the lower extremities to improve gait function according to the intervention schedule. In detail, the training sessions mainly involved strength exercises for leg extensor and flexor muscles as well as muscle groups responsible for leg add- and abduction. Another training aspect focused on proprioceptive training of the lower extremities and trunk stabilizers through balance training in different variations. All exercises were designed to be carried out with the participants’ own individual bodyweight and minimal equipment expenses. Training sessions were supervised via the Zoom conference tool (Zoom Video Communications Inc., San Jose, CA, USA). The long-term aim was to encourage participants to engage in independent exercise, so the supervised session frequency for the IG decreased during the course of the study. Participants exercised twice a week for eight weeks overall. They received two supervised sessions per week in week 1 and 2. In week 3, 4, and 5, they received one supervised session per week and trained unsupervised a second time. This was followed by a period of unsupervised exercise in week 6 and 7. In the last week of the intervention, participants received 2 supervised sessions (intensification phase). Additionally, participants in the IG received a brochure with sport and exercise recommendations for their independent exercise. The control group only received a booklet with general information about physical activity and cancer, but no specific intervention recommendations for patients with endoprosthesis. They were also not encouraged to change their current physical activity behavior during the eight-week study period.

### 2.4. Assessments

To measure the effect of the intervention, all endpoints were assessed at baseline (before the randomization, T0) and after the 8-week intervention/control period (T1 at both appointments (baseline and post-intervention); the assessments were conducted in a standardized order. At first, participants filled in the questionnaires for the subjective rating of physical function, quality of life, and fatigue. For subjective physical function, we used the Musculoskeletal Tumor Society Score (MSTS) and the Toronto Extremity Salvage Score (TESS), which have widely been used for patients with lower extremity musculoskeletal sarcomas [22]. The MSTS scoring system was developed by Enneking et al. [23] and the lower extremity version assesses pain, function, emotional, support, walking, and gait problems on a 0–5 point scale (maximum overall score 30 points). The TESS [24] version for lower extremity sarcomas contains 30 questions to assess physical function in daily activities such as working, dressing, and mobility. Questions are rated on a 1–5 point scale and the total score is calculated as the percentage of the maximum score (leading to a total maximum score of 100 points). Quality of life was assessed with the EORTC QLQ-C30 questionnaire, developed by the European Organization for Research and Treatment of Cancer. It contains 30 questions in subscales (functional score and symptom score) and single items. All subscales and the individual items have a score range from 0 to 100 points. A higher score represents better function and a higher quality of life. However, in the symptom’s subscale, a higher score represents a higher level of symptoms or problems. The EORTC QLQ-FA12 questionnaire was used to evaluate fatigue in the cohort. The EORTC questionnaires are commonly used in cancer patients, including sarcoma patients with lower extremity tumors [25,26]. Participants under the age of 18 years used the Pediatric Quality of Life Inventory (PedsQL) cancer and fatigue modules instead of the EORTC, which have been shown to be valid and reliable in this population [27]. Gait analysis is the most frequently used single physical performance test for bone tumor patients [28]. This was then conducted with a Vicon camera system including 8 cameras recording at 120 Hz at different projections along a runway to record marker positions in a three-dimensional space (Vicon Vantage V5, Oxford, UK). The Conventional Gait Model 2.3 (CGM 2.3) for the lower body, which is also integrated in the Vicon Nexus software, was used to calculate the kinematics of the segments and joints of the lower body of the participants. Each patient walked along a runway (15 m) 5 times in a self-selected walking speed. Five complete gait cycles for the left and right side were selected for further data processing. Gait cycles for each side were defined as the period between the touchdowns of the same foot. The touchdown was defined as the instant when the heel marker reaches the lowest value on the vertical z-coordinate. To assure the greatest possible comparability and minimum variation of intraindividual gait data due to different marker placements at baseline and follow-up measurements, participants’ lower body marker locations were marked after gait analysis with a permanent skin marker. Every participant received a skin marker and was asked to redraw marker locations in case of fading.

After gait analysis, the patients proceeded to the physiological function assessment, which consisted of two functional tests and a knee joint range-of-motion assessment via manual goniometry to assess maximal active and passive knee flexions and extensions. Under consideration of the systematic review by Söntgerath et al. [28] the “timed up and go” test and the “timed up and downstairs” test were conducted. During the “timed up and go”, the participants needed to stand up from a chair, walk a distance of 3 m, return, and sit down again. During the “timed up and downstairs”, participants walked up and down 14 flights of stairs as fast as possible. The endpoint of both tests was the time the participants required to fulfill the task.

### 2.5. Data Analysis and Statistics

To evaluate and analyze the data from the 3D gait analysis in a clinical context, recent studies proposed different indices of overall gait pathology to merge the complex information contained in these highly interdependent 3D data into a single measure [29]. Belonging to the most commonly used gait indices, the Gait Profile Score (GPS) by Baker et al. [29] and the Gait Deviation Index (GDI) by Schwartz and Rozumalski [30] were the two indices used to analyze 3D data in this study. For the calculation of the GPS and GDI scores, the GDI-GPS-Calculator Version3.2 by Richard Baker was used. The different segment angles from the collected gait cycles were normalized over the gait cycle and extracted in 2% increments. For further calculation, gait cycles for each side were averaged. In the study, reference group gait data from healthy subjects (n = 13) in the same age range as the AYA participants were gathered and implemented into the GDI-GPS-Calculator Version 3.2; this was used as the reference dataset to provide valid values using the exact same method, researcher, and biomechanical lab. Four of the 13 healthy subjects were male, nine were female, and the age of the healthy reference group ranged from 22 to 39 years. Further data analysis was carried out using Python data analysis tools (Biomechanical ToolKit, Pandas, NumPy, SciPy, Matplotlib, Seaborn). The differences in the scores of the GPS and GDI between measurements (delta changes) were investigated for differences between CG and IG via an independent *t*-test followed by a calculation of the effect size expressed in Cohen’s d. All changes in patient-reported outcomes as well as the physiological function outcomes between measurements (delta changes) were also compared via an independent t-test, and Cohen’s d was calculated. The significance level for all statistical tests was determined as α = 0.05.

## 3. Results

### 3.1. Patient Characteristics

Eleven participants with lower extremity osteosarcoma around the knee and endoprosthesis, aged between 15 and 41 years, and had implantation surgery at least 12 months before inclusion participated in this RCT. Six participants were allocated to the IG, whereas 5 participants were allocated to the CG (Table 1). No adverse events occurred during gait analysis and the physical performance assessment or during the intervention.

### 3.2. Gait Function at Baseline and Change during the Intervention

**Figure 1:**
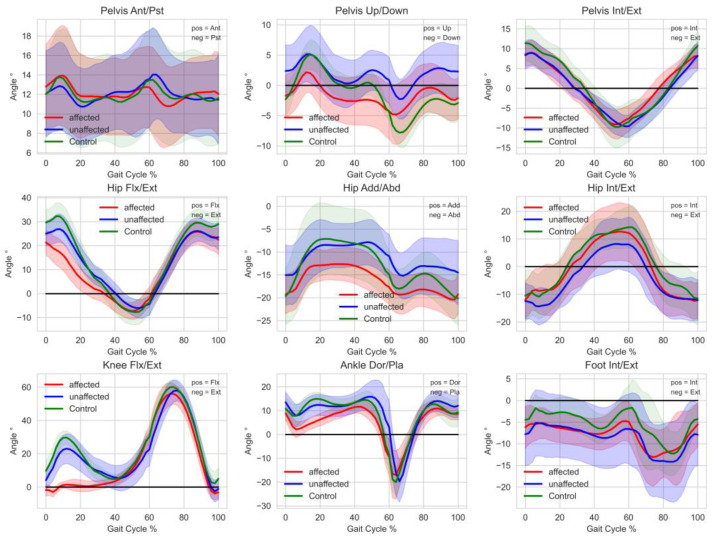
Averaged segment and joint angles throughout the entire gait cycle of all participants at baseline. Unaffected (red) and affected (blue) side of participants with endoprosthesis and a healthy reference group (green) showing the mean and SD (light color area). Healthy reference goup: n = 13, 4 male, 9 female, 22–39 years without tumor megaendoprosthesis.

Gait analysis at baseline revealed that the gait of every participant deviated from a healthy reference group. Deviations were particularly larger in the affected leg than the unaffected leg. This can be seen in individual GDI, GPS, and GVS, as well as the averaged gait curves of all assessed gait variables from all participants throughout the entire gait cycle (see Figure 1). Larger deviations were noticed in the pelvis up/down, hip adduction/abduction, as well as the hip internal/external rotation variables. Furthermore, there were obvious deviations in the knee flexion/extension variable of the affected leg during the first half of the gait cycle in all participants. Summarizing the results of the intervention, Table 2 shows an overview of the relevant descriptive statistical parameters of all assessed outcome measures (gait scores, patient-reported outcomes, and physiological function assessment).

**Figure 2:**
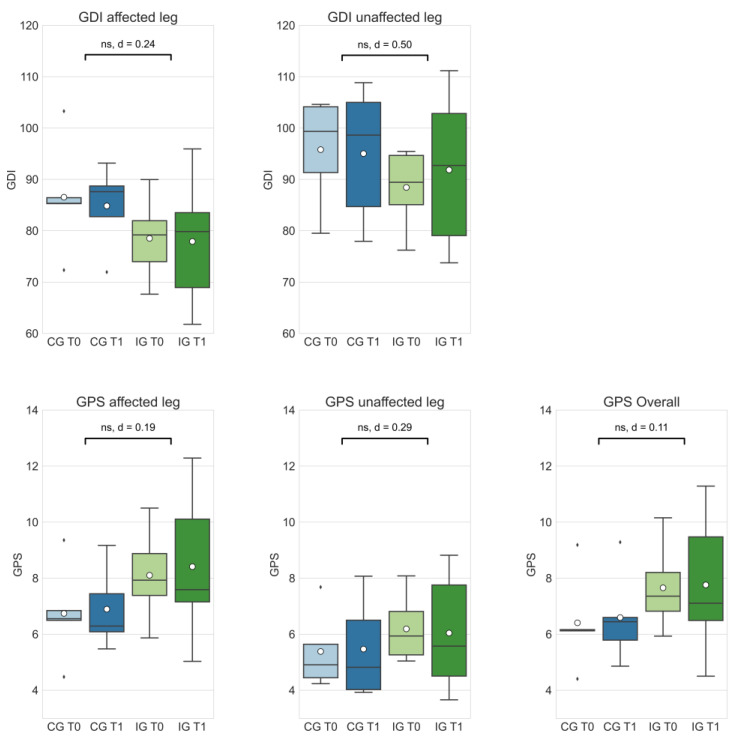
The Gait Deviation Index (GDI) and Gait Profile Score (GPS) of the control group at T0 (CG T0, light blue) and T1 (CG T1, dark blue) as well the intervention group at T0 (IG T0, light green) and T1 (IG T1, dark green). Box-whisker plot shows quartiles, mean (°), and outliers (⬧); statistical annotations show significance (ns = not significant) and effect size (absolute Cohen’s d); comparison of delta changes via independent *t*-test, α = 0.05.

Delta changes between CG and IG were not significant in all assessed gait scores (see Figure 2). Absolute d-values range between |d| = 0.11 (GPS (overall), small effect) and |d| = 0.50 (GDI (unaffected leg), medium effect). Additionally, a larger effect could be identified in the GPS (unaffected leg) variable (|d| = 0.29) compared to the GPS (affected leg) variable (|d| = 0.19).

### 3.3. TESS and MSTS

**Figure 3:**
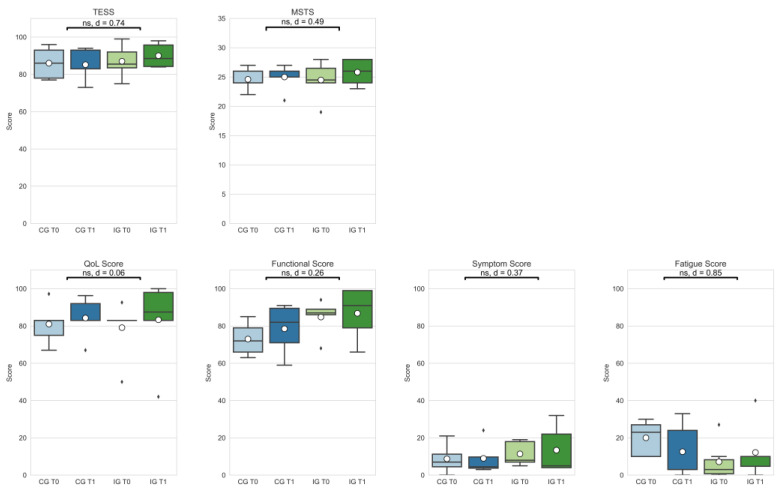
Patient-reported outcomes (Toronto Extremity Salvage Score (TESS); Musculoskeletal Society Score (MSTS); Quality of Life assessment—Function, Symptom, and Fatigue scores) of the control group at T0 (CG T0, light blue) and T1 (CG T1, dark blue) as well as the intervention group at T0 (IG T0, light green) and T1 (IG T1, dark green). Box-whisker plot showing quartiles, mean (°), and outliers (⬧); statistical annotations showing significance (ns = not significant) and effect size (absolute Cohen’s d); comparison delta changes via independent *t*-test, α = 0.05.

Effect sizes ranged from small to large in patient-reported functional outcomes (TESS: |d| = 0.74; MSTS: |d| = 0.49). Delta changes in the patient-reported outcomes did not differ significantly in the comparison between IG and CG (see Figure 3).

### 3.4. Physical Function

**Figure 4:**
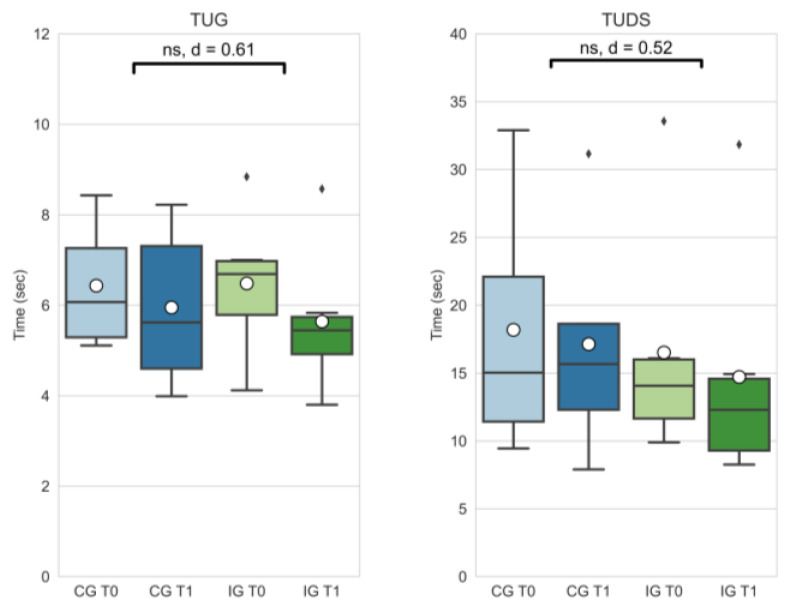
Timed up and go (TUG) and timed up and downstairs (TUDS) outcomes of the control group at T0 (CG T0, light blue) and T1 (CG T1, dark blue) as well as the intervention group at T0 (IG T0, light green) and T1 (IG T1, dark blue). Box-whisker plot showing quartiles, mean (°), and outliers (⬧); statistical annotations showing significance (ns = not significant) and effect size (absolute Cohen’s d); comparison of delta changes via independent *t*-test, α = 0.05.

Effect size for physiological function assessment measures (TUG, TUDS) were medium (TUG: |d| = 0.61; TUDS: |d| = 0.52). Delta changes did not differ significantly (see Figure 4).

### 3.5. Quality of Life and Fatigue

Delta changes in outcomes regarding quality of life showed no significant differences, but small to large effect sizes (Quality of Life (QoL): |d| = 0.06; functional score: |d| = 0.26; symptom score: |d| = 0.37; fatigue score: |d| = 0.85).

## 4. Discussion

The objective kinematic parameters show deviations in the participants’ gait data from healthy control group kinematics and appear on both the affected and unaffected side. This phenomenon seems reasonable because of the necessary interactions between both legs in the gait cycle. Those deviations are comparable to findings of Kim et al. [31], who investigated lower limb joint kinematics of former patients with distal femur and proximal tibia reconstructions. Larger deviations in the pelvic up-and-down motion most likely derive from leg length discrepancies between the affected and unaffected legs. Participants in the proGAIT study had leg length discrepancies ranging from 1.6 to 83.2 mm. These differences are normally compensated through orthopedic devices in the everyday life of participants. Especially in cases with increased LLD (>30 mm), participants usually wear a shoe raise on the affected side. Gait analysis was carried out without compensating footwear to minimize external influences and ensure comparability with the control group data, which were also gathered in barefoot trials. A future option would be a gait analysis involving compensating footwear for participants with increased LLD to allow a detailed look into other potential causes for gait abnormalities.

In addition, larger deviations from healthy gait seem to appear, especially in the knee flexion/extension of the participants’ affected legs during the first half (stance phase to toe-off) of the gait cycle. After the phase of touchdown, the affected knee is nearly fully extended or even overextended in most participants. Compared to knee data from a healthy reference group, which show an initial flexion to about 20–30° after touchdown, this seems to be the most remarkable deviation in participants with endoprosthesis around the knee, which is also mentioned in a paper by Kim et al. [31]. A previous study by Rompen et al. [32] also describes this type of deviation. Some participants of the proGAIT study reported a feeling of instability in the knee when it is slightly bent, so full extension or overextension seems to be a way to compensate for this problem for most of the participants. A possible origin of hyperextension may be the lack of stabilization by the two gastrocnemius muscles, which are always disconnected in distal femoral replacements and at least unilaterally in proximal tibial replacements. The results of a study by Pesenti et al. [10] also suggest that overextension is typical in this cohort. After the touchdown of the affected leg, the quadriceps muscle-tendon units work eccentrically to absorb energy and to decelerate knee flexion. In case of quadriceps weakness or dysfunction after tumor surgery, this is compensated by the hip extensors which then help to bring the lower limb into a more extended position to passively stabilize the knee joint. Thus, hip flexion and extension also deviate in survivors of osteosarcoma with knee tumor endoprosthesis reconstruction compared to a healthy control group. The affected leg especially seems to be more extended at the hip joint throughout the entire gait cycle compared to healthy participants (see Figure 1). Furthermore, proGAIT data show reduced dorsiflexion in the ankle joint compared to the healthy reference group. These deviations seem to appear primarily in the stance phase of the affected leg. Kim et al. [31] hypothesized that this might be due to calf muscle activity stabilizing the tibia to compensate for the above-mentioned quadriceps weakness, and therefore, reduced knee stability. Furthermore, larger deviations can be seen in hip adduction and abduction, and hip internal and external rotation. It remains unclear from where these deviations derive. A meaningful interpretation of outcomes can only be made under the consideration of the gait assessment method, and especially the gait model which was used. The CGM 2.3 still lacks the ability to measure hip rotation and foot progression during dynamic trials with the help of medial markers at the knee and the malleoli, which are used only for the calibration trial. Thus, these results still need to be interpreted with caution.

The results presented in this study indicate positive effects of the intervention in all assessed gait scores, even though the delta differences between groups were statistically not significant (see Figure 2). Larger effects seem to occur in GDI and GPS of patients’ unaffected legs. Due to long periods of physical inactivity [33] and the load restrictions of the affected leg before and after tumor surgery, it can be hypothesized that muscles, and potentially tendon and ligament structures in the unaffected limb, obtain a better trainability, and thus, respond better to an exercise intervention. Medium-to-large positive effects of the intervention can also be seen in patient-reported outcomes, TESS and MSTS (see Figure 3). The review of Kask et al. [22] summarized studies that examined functional outcomes in patients with lower-extremity soft tissue sarcomas and bone sarcomas, and calculated a mean overall TESS score of 86.7, which corresponds to the baseline TESS scores of the proGAIT cohort (CG: 86.0; IG: 87.0). The post-intervention TESS score in the IG group increased to 90.0, which is higher than in previous studies with lower extremity tumor patients [34,35], and indicates a comparably good function.

The objective functional results of the TUG and TUDS also improved in favor of the intervention group (see Figure 4), though these were also not statistically significant. However, an improved functional mobility in daily live facilitates everyday tasks, positively correlates with quality of life [13], and probably also influences physical activity behavior. For most outcomes, both groups showed improvements after the study period of 8 weeks. The IG, however, showed a decline in outcomes in the symptom and fatigue scale. Although in contrast with the general evidence regarding the effectiveness of exercise to reduce fatigue in cancer patients [16], for some patients, the transition to strenuous exercise may have resulted in an increased workload that was unfamiliar and also fatiguing for them. It can also be hypothesized that the duration of the intervention was too short, and that the results would be different if the supervision was in person and not via an online platform. It should be clear that an exercise intervention for this patient group should be well balanced and individually adjusted to avoid excessive physical load, especially on structures affected by former tumor resection surgery. Furthermore, previous chemotherapy treatment and corresponding late effects should be considered in the exercise planning. Improvements in the above-mentioned outcomes in both study groups are probably a result of the contamination of the CG. This is a well-known problem in exercise oncology studies, as described by Steins Bisschop et al. [36]. The CG received a booklet with general information about physical activity and cancer prior to the intervention period. Participation in gait analysis seemed to create awareness of the issue and potentially motivated participants to increase their physical activity and lower limb function.

The systematic review by Wilson et al. [37] did not identify any studies involving an exercise intervention in combination with an objective gait assessment in this context, although improvement in lower limb function, and thus, quality of life, is becoming an increasing issue for this patient group. A long-term prospective study by Egmond-van-Dam et al. [38] with former bone tumor patients investigated long-term functional outcomes, focusing on the knee joint. They found no significant changes in functional outcome measures between 2 and 7 years after surgery. It, therefore, seems appropriate to test the effectiveness of an intervention that starts earlier.

The proGAIT study has several limitations. The overall sample size is small, so that even in the case of medium-to-large effect sizes, no statistical significance could be detected. Furthermore, the gait assessment time from surgery was different between participants and might be a reason for the varying effects of the exercise intervention in different participants. It could not be guaranteed that the markers were placed in exactly the same place before and after the intervention. However, intensive palpation seemed to have led to the correct placement. Some differences between IG and CG characteristics were noted that seem be present as a result of the small sample size. In the IG, half of participants had a history of proximal tibia tumor versus 20% in the CG. IG participants were younger at surgery, had a longer follow-up period since surgery, and had larger leg lengths discrepancies. However, since all intervention effects were calculated as the change to baseline, these clinical differences seem to be negligible in terms of outcomes. Another limitation regarding gait analysis and comparability with the above-mentioned studies is the lack of kinetic outcomes in gait analysis. This would have been an option to gain a more detailed look into typical gait deviations in the investigated group of former cancer patients.

## 5. Conclusions

The exploratory proGAIT randomized controlled trial shows promising positive effects—however, without statistical significance—of an 8-week exercise intervention on lower limb gait function in former patients with tumor endoprostheses around the knee. Additional powered clinical trials with larger samples sizes are needed to confirm these preliminary results. In a subsequent study, further questions need to be addressed, such as the ideal time after surgery to start the supervised gait exercise program, the most effective exercises in the program, and the preferred intensity and duration. Nonetheless, it should be noticed that exercise has already been shown to be beneficial for several groups of cancer patients and survivors, and existing guidelines in pediatric and adult oncology provide feasible and safe concepts to improve the mobility and physical performance and reduce symptoms [16,39,40]. Therefore, in addition to the required confirmatory studies, there is a particular need for the development of implementation approaches, such as the partially supervised online training from proGAIT. The long-term goal is to implement effective exercise concepts as the standard care for AYA survivors with a tumor endoprosthesis and survivors with other consequences of cancer treatment.

## Figures and Tables

**Table 1:** Demographic characteristics of the study population at baseline.

		IG			CG	
Characteristic	Mean	SD	Range	Mean	SD	Range
Number of patients	6	-	-	5	-	-
Male/female	3/3	-	-	3/2	-	-
Tumor location						
(Proximal tibia/distal femur)	3/3	-	-	1/4	-	-
Age at gait analysis (years)	26.3	8.0	15–34	27.0	9.8	17–41
Age at surgery (years)	19.8	7.5	12–31	24.0	11.2	10–39
Follow-up (years)	6.5	6.1	1–16	3.0	2.3	1–7
Weight (kg)	69	12.4	52.0–82.0	76.1	25.9	60.0–122.0
Height (cm)	172.2	7.7	163–182	179.0	4.7	172.0–185.0
BMI (kg/m^2^)	23.4	4.7	18.0–29.1	23.4	6.8	20.0–35.6
Leg Length Discrepancy (mm)	23.7	30.5	1.6–83.2	13.6	13.3	3.9–36.9

IG, intervention group; CG, control group; SD, standard deviation.

**Table 2:** Overview of the relevant descriptive statistical parameters of all assessed outcome measures at baseline (T0) and after the intervention (T1).

			GDI (aff)	GDI (unaff.)	GPS (aff.)	GPS (unaff.)	GPS (Overall)	TESS	MSTS	QoL	Function	Symptom	Fatigue	TUG (sec)	TUDS (sec)	Knee Flexion Active (°)	Knee Flexion Passive (°)	Knee Extension Active (°)	Knee Extension Passive (°)
CG (n = 5)	T0	Mean	86.5	95.8	6.7	5.4	6.4	86.0	24.6	81.0	73.0	8.75	20.0	6.4	18.2	90.7	92.4	13.3	0.0
		Min	72.3	79.5	4.5	4.2	4.4.	77.0	22.0	67.0	63.0	0.0	10.0	5.1	9.5	80.0	85.0	5.0	0.0
		Max	103.3	104.6	9.4	7.7	9.2	96.0	27.0	97.2	85.0	21.0	30.0	8.4	32.9	105.0	110.0	30.0	0.0
		95%-CI	72.9–100.2	82.7–108.9	4.6–8.9	3.6–7.1	4.3–8.6	75.4–96.6	22.2–27.0	67.1–95.0	57.2–88.8	0.0–22.8	8.3–31.7	4.7–8.2	6.3–30.0	58.6–122.7	79.9–104.9	0.0–49.2	-
	T1	Mean	84.8	95.0	6.9	5.5	6.6	85.2	25.0	84.3	78.5	9.0	12.6	5.9	17.1	86.0	95.2	14.0	2.0
		Min	71.9	78.0	5.5	3.9	4.9	73.0	21.0	67.0	59.0	3.0	0.0	4.0	7.9	70.0	85.0	0.0	0.0
		Max	93.2	108.8	9.1	8.1	9.3	94.	27.0	96.3	91.0	24.0	33.0	8.2	31.2	108.0	115.0	45.0	10.0
		95%-CI	74.8–94.9	78.6–111.5	5.1–8.7	3.3–7.7	4.5–8.6	74.5–95.9	22.1–28.0	70.3–98.2	54.9–100.0	0.0–25.0	0.0–31.1	3.7–8.1	6.2–28.1	68.9–103.1	79.7–110.7	0.0–36.1	0.0–7.6
IG (n = 6)	T0	Mean	78.5	88.4	8.1	6.2	7.7	87.0	24.5	79.1	84.8	11.4	7.2	6.5	16.5	87.5	97.5	26.3	1.7
		Min	67.6	76.2	5.9	5.0	5.9	75.0	19.0	50.0	68.0	5.0	0.0	4.1	9.9	66.0	70.0	10.0	0.0
		Max	90.0	95.4	10.5	8.1	10.2	99.0	28.0	92.6	94.0	19.0	27.0	8.8	33.6	110.0	120.0	50.0	10.0
		95%-CI	70.4–86.7	80.5–96.4	6.4–9.8	5.0–7.4	6.1–9.2	78.1–95.9	21.2–27.8	63.6–94.6	72.5–97.1	3.2–19.6	0.0–18.1	4.8–8.1	7.4–25.6	72.3–102.7	78.2–116.8	7.6–45.1	0.0–6.0
	T1	Mean	77.9	91.8	8.4	6.0	7.8	90.0	25.8	83.3	86.8	13.4	12.2	5.6	14.7	82.5	98.3	16.0	0.8
		Min	61.8	73.8	5.0	3.7	4.5	84.0	23.0	42.0	66.0	4.0	0.0	3.8	8.3	55.0	70.0	0.0	0.0
		Max	96.0	111.2	12.3	8.8	11.3	98.0	28.0	100.0	99.0	32.0	40.0	8.6	31.8	120.0	125.0	45.0	5.0
		95%-CI	64.8–91.0	75.8–107.9	5.6–11.2	3.8–8.3	5.1–10.4	83.1–96.9	23.3–28.4	60.6–100.0	69.1–100.0	0.0–29.4	0.0–27.2	4.0–7.3	5.5–23.9	59.8–105.2	75.2–121.4	0.0–37.2	0.0–3.0

Gait Deviation Index (GDI), Gait Profile Score (GPS), Toronto Extremity Salvage Score (TESS); Musculoskeletal Tumor Society Score (MSTS); Quality of Life assessment (QoL), Function, Symptom and Fatigue Scale; Timed up and go (TUG); Timed up and downstairs (TUDS). Mean values (mean), min. values (min), max. values (max), and 95% confidence intervals (95%-CI); aff = affacted; unaff = unaffected (without prosthesis); sec = seconds; ° = angle degree.

## Data Availability

The data are available from the corresponding author on reasonable request.

## References

[1] Miller K.D., Fidler-Benaoudia M., Keegan T.H., Hipp H.S., Jemal A., Siegel R.L. (2020). Cancer statistics for adolescents and young adults, 2020. CA A Cancer J. Clin..

[2] Meltzer P.S., Helman L.J. (2021). New Horizons in the Treatment of Osteosarcoma. N. Engl. J. Med..

[3] Bölling T., Hardes J., Dirksen U. (2013). Management of bone tumours in paediatric oncology. Clin. Oncol. (R. Coll. Radiol.).

[4] Grünewald T.G.P., Cidre-Aranaz F., Surdez D., Tomazou E.M., de Álava E., Kovar H., Sorensen P.H., Delattre O., Dirksen U. (2018). Ewing sarcoma. Nat. Rev. Dis. Primers.

[5] Pugh G., Gravestock H.L., Hough R.E., King W.M., Wardle J., Fisher A. (2016). Health Behavior Change Interventions for Teenage and Young Adult Cancer Survivors: A Systematic Review. J. Adolesc. Young Adult Oncol..

[6] Pugh G., Hough R., Gravestock H., Davies C., Horder R., Fisher A. (2019). The development and user evaluation of health behaviour change resources for teenage and young adult Cancer survivors. Res. Involv. Engagem..

[7] Pedersen C., Rechnitzer C., Andersen E.A.W., Kenborg L., Norsker F.N., Bautz A., Baad-Hansen T., Tryggvadottir L., Madanat-Harjuoja L.-M., Holmqvist A.S. (2021). Somatic Disease in Survivors of Childhood Malignant Bone Tumors in the Nordic Countries. Cancers.

[8] Ranft A., Seidel C., Hoffmann C., Paulussen M., Warby A.-C., Berg H.V.D., Ladenstein R., Rossig C., Dirksen U., Rosenbaum D. (2017). Quality of Survivorship in a Rare Disease: Clinicofunctional Outcome and Physical Activity in an Observational Cohort Study of 618 Long-Term Survivors of Ewing Sarcoma. J. Clin. Oncol..

[9] Tan P., Yong B., Wang J., Huang G., Yin J., Zou C., Xie X., Tang Q., Shen J. (2012). Analysis of the efficacy and prognosis of limb-salvage surgery for osteosarcoma around the knee. Eur. J. Surg. Oncol..

[10] Pesenti S., Peltier E., Pomero V., Authier G., Roscigni L., Viehweger E., Jouve J.-L. (2018). Knee function after limb salvage surgery for malignant bone tumor: Comparison of megaprosthesis and distal femur allograft with epiphysis sparing. Int. Orthop..

[11] Mester B., Guder W., Streitbürger A., Schoepp C., Nottrott M., Podleska L., Dudda M., Hardes J. (2021). Wiederkehr zu körperlicher Aktivität und Sport in der Tumororthopädie. [Return to Sports and Activity in Tumor Orthopaedics]. Z. Orthop. Unfall..

[12] Gerber L.H., Hoffman K., Chaudhry U., Augustine E., Parks R., Bernad M., Mackall C., Steinberg S., Mansky P. (2006). Functional outcomes and life satisfaction in long-term survivors of pediatric sarcomas. Arch. Phys. Med. Rehabil..

[13] Liu Y., Hu A., Zhang M., Shi C., Zhang X., Zhang J. (2014). Correlation between functional status and quality of life after surgery in patients with primary malignant bone tumor of the lower extremities. Orthop. Nurs..

[14] Bekkering W.P., Vlieland T.V., Koopman H.M., Schaap G.R., Schreuder H.B., Beishuizen A., Tissing W.J., Hoogerbrugge P.M., Anninga J.K., Taminiau A.H. (2010). Quality of life in young patients after bone tumor surgery around the knee joint and comparison with healthy controls. Pediatr. Blood Cancer.

[15] Müller D.A., Beltrami G., Scoccianti G., Cuomo P., Capanna R. (2016). Allograft-prosthetic composite versus megaprosthesis in the proximal tibia-What works best?. Injury.

[16] Campbell K.L., Winters-Stone K.M., Wiskemann J., May A.M., Schwartz A.L., Courneya K.S., Zucker D.S., Matthews C.E., Ligibel J.A., Gerber L.H. (2019). Exercise Guidelines for Cancer Survivors: Consensus Statement from International Multidisciplinary Roundtable. Med. Sci. Sports Exerc..

[17] Patel A.V., Friedenreich C.M., Moore S.C., Hayes S.C., Silver J.K., Campbell K.L., Winters-Stone K., Gerber L.H., George S.M., Fulton J.E. (2019). American College of Sports Medicine Roundtable Report on Physical Activity, Sedentary Behavior, and Cancer Prevention and Control. Med. Sci. Sports Exerc..

[18] Winter C.C., Müller C., Hardes J., Gosheger G., Boos J., Rosenbaum D. (2013). The effect of individualized exercise interventions during treatment in pediatric patients with a malignant bone tumor. Support. Care Cancer.

[19] Choi E., Becker H., Kim S. (2022). Unmet needs in adolescents and young adults with cancer: A mixed-method study using social media. J. Pediatr. Nurs..

[20] Saghaei M., Saghaei S. (2011). Implementation of an open-source customizable minimization program for allocation of patients to parallel groups in clinical trials. JBiSE.

[21] Westlake B., Pipitone O., Tedesco N.S. (2022). Time to Functional Outcome Optimization After Musculoskeletal Tumor Resection. Cureus.

[22] Kask G., Barner-Rasmussen I., Repo J.P., Kjäldman M., Kilk K., Blomqvist C., Tukiainen E.J. (2019). Functional Outcome Measurement in Patients with Lower-Extremity Soft Tissue Sarcoma: A Systematic Literature Review. Ann. Surg. Oncol..

[23] Enneking W.F., Dunham W., Gebhardt M.C., Malawar M., Pritchard D.J. (1993). A system for the functional evaluation of reconstructive procedures after surgical treatment of tumors of the musculoskeletal system. Clin. Orthop. Relat. Res..

[24] Davis A.M., Wright J.G., Williams J.I., Bombardier C., Griffin A., Bell R.S. (1996). Development of a measure of physical function for patients with bone and soft tissue sarcoma. Qual. Life Res..

[25] Reijers S.J., Husson O., Soomers V.L., Been L.B., Bonenkamp J.J., van de Sande M.A., Verhoef C., van der Graaf W.T., van Houdt W.J. (2022). Health-related quality of life after isolated limb perfusion compared to extended resection, or amputation for locally advanced extremity sarcoma: Is a limb salvage strategy worth the effort?. Eur. J. Surg. Oncol..

[26] Saebye C., Fugloe H.M., Nymark T., Safwat A., Petersen M.M., Baad-Hansen T., Krarup-Hansen A., Keller J. (2017). Factors associated with reduced functional outcome and quality of life in patients having limb-sparing surgery for soft tissue sarcomas—A national multicenter study of 128 patients. Acta Oncol..

[27] Varni J.W., Burwinkle T.M., Katz E.R., Meeske K., Dickinson P. (2002). The PedsQL in pediatric cancer: Reliability and validity of the Pediatric Quality of Life Inventory Generic Core Scales, Multidimensional Fatigue Scale, and Cancer Module. Cancer.

[28] Söntgerath R., Däggelmann J., Kesting S.V., Rueegg C.S., Wittke T.-C., Reich S., Eckert K.G., Stoessel S., Chamorro-Viña C., Wiskemann J. (2021). Physical and functional performance assessment in pediatric oncology: A systematic review. Pediatr. Res..

[29] Baker R., McGinley J.L., Schwartz M.H., Beynon S., Rozumalski A., Graham H.K., Tirosh O. (2009). The gait profile score and movement analysis profile. Gait Posture.

[30] Schwartz M.H., Rozumalski A. (2008). The Gait Deviation Index: A new comprehensive index of gait pathology. Gait Posture.

[31] Kim S., Ryu C., Jung S.-T. (2021). Differences in Kinematic and Kinetic Patterns According to the Bone Tumor Location after Endoprosthetic Knee Replacement Following Bone Tumor Resection: A Comparative Gait Analysis between Distal Femur and Proximal Tibia. J. Clin. Med..

[32] Rompen J.C., Ham S.J., Halbertsma J.P.K., van Horn J.R. (2002). Gait and function in patients with a femoral endoprosthesis after tumor resection: 18 patients evaluated 12 years after surgery. Acta Orthop. Scand..

[33] Winter C., Müller C., Brandes M., Brinkmann A., Hoffmann C., Hardes J., Gosheger G., Boos J., Rosenbaum D. (2009). Level of activity in children undergoing cancer treatment. Pediatr. Blood Cancer.

[34] Bamdad K., Hudson S., Briggs T. (2019). Factors associated with functional outcome in patients having limb salvage surgery for primary malignant bone sarcoma using TESS. J. Clin. Orthop. Trauma.

[35] Heaver C., Isaacson A., Gregory J.J., Cribb G., Cool P. (2016). Patient factors affecting the Toronto extremity salvage score following limb salvage surgery for bone and soft tissue tumors. J. Surg. Oncol..

[36] Bisschop C.N.S., Courneya K.S., Velthuis M.J., Monninkhof E.M., Jones L.W., Friedenreich C., van der Wall E., Peeters P.H.M., May A.M. (2015). Control group design, contamination and drop-out in exercise oncology trials: A systematic review. PLoS ONE..

[37] Wilson P.J., Steadman P., Beckman E.M., Connick M.J., Carty C.P., Tweedy S.M. (2019). Fitness, Function, and Exercise Training Responses after Limb Salvage with a Lower Limb Megaprosthesis: A Systematic Review. PM&R.

[38] Van Egmond-van Dam J.C., Bekkering W.P., Bramer J.A.M., Beishuizen A., Fiocco M., Dijkstra P.D.S. (2017). Functional outcome after surgery in patients with bone sarcoma around the knee; results from a long-term prospective study. J. Surg. Oncol..

[39] Wurz A., McLaughlin E., Lategan C., Viña C.C., Grimshaw S.L., Hamari L., Götte M., Kesting S., Rossi F., van der Torre P. (2021). The international Pediatric Oncology Exercise Guidelines (iPOEG). Transl. Behav. Med. Soc. Behav. Med..

[40] Götte M., Gauß G., Dirksen U., Driever P.H., Basu O., Baumann F.T., Wiskemann J., Boos J., Kesting S.V. (2022). Multidisciplinary Network ActiveOncoKids guidelines for providing movement and exercise in pediatric oncology—Consensus-based recommendations. Pediatr. Blood Cancer.

